# HCV-infected Renal Transplant Recipients: Our Experience before the Availability of New Antiviral Drugs

**Published:** 2017-05-01

**Authors:** A. R. Fernandes, I. J. Laranjinha, R. Birne, P. Matias, C. Jorge, T. Adragão, M. Bruges, A. Weigert, D. Machado

**Affiliations:** 1 Department of Nephrology, Hospital São Bernardo, Setúbal, Portugal; 2 Department of Nephrology, Hospital Santa Cruz, Carnaxide, Portugal

**Keywords:** Hepatitis C, Kidney transplantation, Graft rejection, Survival Rate, Transplant recipients, Postoperative complications

## Abstract

**Background:**

Natural history of HCV-infected renal transplant recipients is about to change with the invention of new drugs available for the treatment of HCV.

**Objective:**

To analyze the evolution of renal transplant recipients infected with HCV in 30 years of activity of a Renal Transplantation Unit.

**Methods:**

We studied 1334 patients who underwent renal transplantation between 1985 and 2015.

**Results:**

189 (14.2%) of these 1334 were found HCV seropositive. 60 were HCV RNA-positive for >6 months. 5 died with a functioning graft; 19 lost their graft and resumed dialysis. Most of the rejections occurred within the first year of the transplantation and none resulted in immediate loss of the graft. In post-transplantation period, 14 patients developed clinical hepatic disease, 10 manifested new-onset diabetes after transplantation, and 4 had *de novo* neoplasia, none of them had hepatocellular carcinoma. The outcomes of the different variables analyzed were similar between patients with HCV-infection and those with HCV and HBV co-infection. The median survival time was 13.4 (95% CI: 10.7–16.1) years; the median survival time of patients without HCV infection was 14.6 (95% CI: 13.8–15.4) years (p=0.23).

**Conclusion:**

In the era before the availability of new anti-HCV drugs, our experience with HCV-infected renal transplant recipients revealed similar post-transplantation complications, graft and patient survival as those not infected with HCV.

## INTRODUCTION

Hepatitis C virus (HCV) affects approximately 170 million people worldwide; It is also being a major cause of end-stage liver disease leading to liver transplantation [1, 2]. The prevalence of HCV infection in patients with chronic kidney disease (CKD) exceeds that in the general population [3]. In the past, blood transfusion has played a major role in the transmission of HCV to patients under chronic hemodialysis [4]. There has been a noticeable decrease in HCV infection among these patients, however, after the introduction of regular screening for HCV and the use of erythropoietin [5].

HCV not only can lead to kidney disease [6–12], but also contributes to increased morbidity and mortality in patients with established CKD [13]. Kidney transplantation is recognized as the renal replacement therapy of choice for eligible patients with end-stage renal disease (ESRD) [14]. Some studies have shown better survival in HCV-positive kidney transplant recipients than in HCV-positive patients with ESRD who are on dialysis [15]. The optimal immunosuppressive regimen in this group of patients remains uncertain [15].

Early detection, prevention, and treatment of complications caused by chronic HCV infection may improve the outcomes of infected kidney transplant recipients [16]. Screening for HCV infection is done using a serological assay that detects antibodies against HCV (anti-HCV). After a positive anti-HCV test, HCV RNA testing should be performed. A negative result is considered a resolved HCV infection (or a false-positive antibody test) [17].

Until the introduction of new anti-HCV drugs, some authors suggested that all kidney transplant candidates should undergo antiviral treatment before receiving a kidney transplant [18]. As high efficiency therapy started to be used in our patients, we decided to review the evolution of HCV patients prior to availability of these drugs. We therefore conducted a retrospective study to determine the follow-up of kidney transplant recipients in our center.

## PATIENTS AND METHODS

We included the patients transplanted from May 1985 to November 2015. During this period, there were 1334 kidney transplantations in our center, the António Pina Renal Transplant Unit. Those with a positive HCV RNA for more than 6 months were considered “HCV infected.” Although the study was started in 1985, the qualitative research of HCV started on 1995 and the viral load was determined in 1997.

The following variables were studied: donor age and HCV status, recipient age and sex, duration of hemodialysis, pre- and post-transplantation liver status, HBV and HIV co-infection as well as delayed graft function, immunosuppression, frequency of biopsy-proven rejection (acute and chronic, and cellular and humoral), mean serum creatinine at one year, frequency of new-onset diabetes after transplantation (NODAT), bacteremia, cytomegalovirus infection, cardiovascular events, neoplasia, patient and graft survival in both groups—with and without HCV infection. 

Statistical Analysis

Statistical analyses were performed with SPSS ver 22.0 (IBM, IBM Corporation, Armonk, NY, USA). Results were expressed as frequencies and percentages, and mean±SD or median (IQR), as appropriate. Graft and patient survival rates were assessed using the Kaplan-Meier survival analysis. 

## RESULTS

HCV antibodies were found in 189 recipients; only 60 had chronic infection, determined by the presence of HCV RNA. Patient demographics are reported in Table 1. Only one patient had pre-transplantation clinical hepatic disease.

**Table 1:** Patient demographics

Parameter	Statistics
HCV-positive recipients	60
Mean±SD age (yrs)	41.7±16.8
Male Female	40 20
Caucasian African	51 9
Donor type	
Living Deceased Missing data	4 53 3
Median (IQR) dialysis time (m)	79.4 (38.2– 158.0)
Prior transplantation	11
PRA>60%	39
Cause of ESRD	
Diabetes Hypertension Glomerulonephritis Unknown Others	2 4 5 15 34
Median (IQR) follow-up (m)	112.3 (109.5–247.0)
Pre-transplant clinical hepatic disease	1
HBV co-infection	7
HIV co-infection	0
HCV genotype 1A 1B 2 3 4	16 28 2 7 7

Two-thirds of the patients were male. The mean±SD age of studied recipients was 41.7±16.8 years. Fifty-four patients received the transplant from a deceased donor. All donors had negative antibodies against HCV. The median time in dialysis was 79 months; only one patient had clinical liver disease before the transplantation. This patient had HCV and HBV co-infection. Forty-four patients had HCV genotype 1. 

Seven patients had also HBV co-infection, of whom the genotype distribution was one with genotype 1A, four with 1B, one with genotype 3, and another one with genotype 4.

The initial immunosuppressive treatment is listed in Table 2. Most of the patients maintained the immunosuppression with a calcineurin inhibitor; 16 (27%) of 60 changed the immunosuppressive regimen; 11 (18%) switched to an mTOR inhibitor.

**Table 2:** Initial immunosuppressive therapy

Regimen	n
ATG + MMF + MPD + CSA	8
ATG + MMF + MPD + TAC	8
AZA + TAC	4
Basiliximab + MMF + CSA + MPD	2
Basiliximab + MMF + TAC + MPD	9
CSA + AZA + MPD	14
CSA + MMF + MPD	8
Missing	7

ATG: thymoglobulin, MMF: micophenolate mofetil, MPD: methylprednisolone, CSA: cyclosporine, TAC: tacrolimus,AZA: azathioprine

Fourteen (23%) of HCV-infected patients developed clinical hepatic disease after renal transplantation.

Causes of graft failure were chronic allograft nephropathy (n=8), vascular or urological problems (n=2), undetermined (n=8), and membranous glomerulonephritis (n=1).

Death with a functioning graft (DWFG) occurred in five (8%) patients after a median follow-up of 318 (IQR: 292–341) months post-transplantation. Causes of DWFG were infection (n=2), acute liver failure (n=1), and undetermined (n=2). Two of the DWGF developed clinical hepatic disease in the post-transplantation period.

Biopsy-proven acute rejection occurred in 10 (17%) patients within the first year, and in 7 (12%) thereafter. Of the patients with rejection, three ultimately developed graft failure. The mean time to graft failure was 215 (range: 197–233) months (Table 3).

**Table 3:** Complications during the first year post-transplantation

Good initial function	40
Acute tubular necrosis (ATN)	14
Mean±SD duration of ATN (days)	4±7
First-year rejections	10
First-year rejections (episodes/patient)	0.17
First-year mean creatinine level (mg/dL)	1.33

Several other outcomes were of clinical interest. New-onset diabetes after transplantation occurred in 10 (17%) recipients. Cardiovascular events occurred in 3 (5%) patients. Seven (12%) had cytomegalovirus infection, and four (7%) patients developed neoplasia during the follow-up period, none of which was hepatocellular carcinoma (Table 4).

**Table 4:** Post-transplantation complications

Complication	n
Acute rejection after the first year	
Cellular Humoral	4 3
Cardiovascular events	3
Neoplasia	4
Bacteriemia	5
Cytomegalovirus	7
Abnormal levels of liver enzymes	4
NODAT	10
Post-transplantation clinical hepatic disease	14

**Figure 1:**
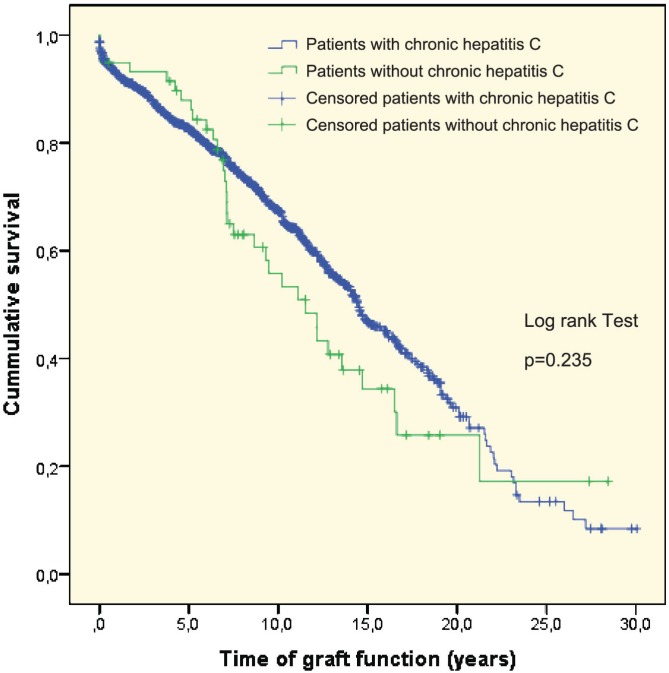
Kaplan-Meier curve demonstrating graft survival

**Figure 2:**
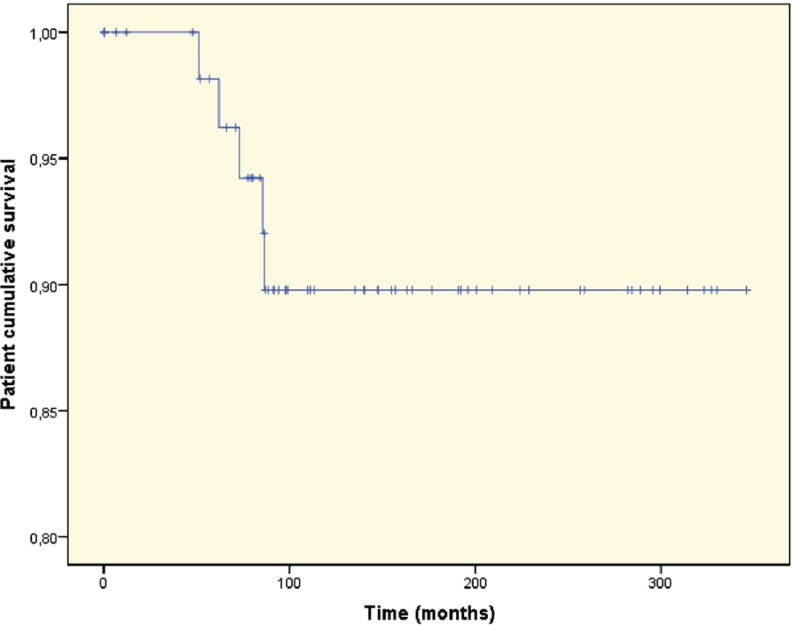
Kaplan-Meier curve demonstrating patient survival

Of the seven patients transplanted with HCV and HBV co-infection, one developed NODAT and one developed neoplasia. The median survival time was 13.4 years. The median graft survival time was 13.4 (95% CI: 10.7–16.1) years. The median graft survival for those patients without HCV infection was 14.6 (95% CI: 13.8–15.4) years (p=0.23) (Fig 1). Figure 2 shows the Kaplan-Meier curve of patient survival time, though this may be biased due to lost to follow up of survived patients who lost the graft.

## DISCUSSION

We reviewed a broad period that has witnessed major changes in the transplantation medicine. In the early years of the study, a sizable amount of patients became infected with HCV due to high transfusion need and lack of efficient HCV screening in blood products. Our patients had a median dialysis time of 79 months that could probably explain how they acquired the infection.

Due to the fact that the determination of HCV viral load started only 12 years after the beginning of the study, we might have excluded some patients from the analysis, because the status of chronic hepatitis C was unknown. 

The natural history of the HCV hepatitis in post-renal transplantation has not been well established. Some authors believe that the immunosuppressive therapy facilitates viral proliferation and aggravates liver disease [19, 20]. However, an increase in viremia may not be associated with a higher risk of liver disease after the renal transplantation [21, 22].

Patients with HCV infection are prone to hepatic and extra-hepatic complications after kidney transplantation. we designed the current study to understand the complications that our patient developed in the post-transplantation period. HCV infection is the leading cause of liver disease after kidney transplantation and is associated with an increase in mortality [23]. Chronic hepatitis and its sequelae are the main forms of liver disease in these patients. HCV-induced cirrhosis is associated with a high risk of hepatocellular carcinoma [17]. The incidence of hepatocellular carcinoma may be higher in kidney transplant recipients compared to the general population [24]. In our sample 14 (23%) HCV-infected patients developed clinical hepatic disease, which was in line with the literature. None had progressed to hepatocellular carcinoma.

The risk factors that were most consistently associated with progression of fibrosis were severity of liver disease before transplantation and duration of follow-up after transplantation [25]. In our series, the patients who developed clinical hepatic disease had longer follow ups.

Extra-hepatic complications of HCV infection after renal transplantation include glomerulonephritis, NODAT, infection, and neoplasia.

There is strong epidemiological, clinical, and experimental data linking chronic HCV infection to glomerulonephritis in native as well as transplanted kidneys [26-28]. Both in native kidneys and in kidney allografts, HCV may cause a variety of glomerular patterns. Membranoproliferative glomerulonephritis is the most common condition and is sometimes difficult to distinguish from chronic allograft nephropathy. Eight of the 19 lost allografts in our series had histological evidence of chronic allograft nephropathy. The role of HCV is uncertain in each case. One patient in our study lost his graft due to glomerulopathy, specifically *de novo* membranous glomerulonephritis, which also can be linked to the virus.

HCV infection has been associated with insulin resistance [29, 30], and diabetes mellitus in the general population [31-33]. Some authors have shown that chronic HCV infection is an independent risk factor for NODAT as well, both in kidney and in liver transplant recipients [17]. Ten (17%) patients developed NODAT.

Several early studies found a significantly increased risk for other infections in HCV-infected recipients [34-36], with the highest risk in the first 6–12 months post-transplantation. That was not evident in our study.

A meta-analysis found a 5.7-fold increase in risk for non-Hodgkin lymphoma in HCV-infected patients [37]. Four of our patients developed neoplasia post-transplantation, but none of them had developed lymphoma or hepatocellular carcinoma.

In this miscellaneous group of patients transplanted from 1985 to 2015, the median survival time of the kidney allograft was 13.4 (95% CI: 10.7–16.1). The median survival time of the graft in the control group without HCV infection was 14.6 (95% CI: 13.8–15.4) years, p=0.23.

In conclusion, HCV infection has long been recognized as the main liver disease in kidney transplantation. Despite the lack of efficacious and safe therapies for HCV in kidney transplant recipients until recently, during the study period we had satisfactory outcomes. This experience can be used for future comparisons with studies in the era after the introduction of new antiviral drugs now available.

## CONFLICTS OF INTEREST:

**None declared.**
